# Fabrication of biodegradable chicken feathers into ecofriendly-functionalized biomaterials: characterization and bio-assessment study

**DOI:** 10.1038/s41598-022-23057-4

**Published:** 2022-10-31

**Authors:** Doaa A. Goda, Mohamed A. Diab, Hamada El-Gendi, Elbadawy A. Kamoun, Nadia A. Soliman, Ahmed K. Saleh

**Affiliations:** 1 Bioprocess Development Department, Genetic Engineering and Biotechnology Research Institute (GEBRI), City of Scientific Research and Technological Applications (SRTA-City), New Borg El-Arab City, Universities and Research Institutes Zone, P.O. 21934, Alexandria, Egypt; 2 Cellulose and Paper Department, National Research Centre, 33 El-Bohouth St., Dokki, P.O. 12622, Giza, Egypt; 3 Nanotechnology Research Center (NTRC), The British University in Egypt (BUE), El-Sherouk City, P.O. 11837, Cairo, Egypt; 4 Polymeric Materials Research Dep. Advanced Technology and New Materials Research Institute (ATNMRI), City of Scientific Research and Technological Applications (SRTA-City), New Borg Al-Arab City, 21934, Alexandria, Egypt

**Keywords:** Biotechnology, Microbiology

## Abstract

This study aims to investigate novel applications for chicken feather waste hydrolysate through a green, sustainable process. Accordingly, an enzymatically degraded chicken feather (EDCFs) product was used as a dual carbon and nitrogen source in the production medium of bacterial cellulose (BC). The yield maximization was attained through applying experimental designs where the optimal level of each significant variable was recorded and the yield rose 2 times. The produced BC was successfully characterized by FT-IR, XRD and SEM. On the other hand, sludge from EDCFs was used as a paper coating agent. The mechanical features of the coated papers were evaluated by bulk densities, maximum load, breaking length, tensile index, Young’s modulus, work to break and coating layer. The results showed a decrease in tensile index and an increase in elongation at break. These indicate more flexibility of the coated paper. The coated paper exhibits higher resistance to water vapor permeability and remarkable oil resistance compared to the uncoated one. Furthermore, the effectiveness of sludge residue in removing heavy metals was evaluated, and the sorption capacities were ordered as Cu ++  > Fe ++  > Cr ++  > Co ++ with high affinity (3.29 mg/g) toward Cu ++ and low (0.42 mg/g) towards Co ++ in the tested metal solution.

## Introduction

Annual production of feathers from the world's chicken sector is estimated to be millions of tonnes^1^**.** Feather waste is relatively new natural, available and zero cost when compared with other wastes. It’s considered the major and abundant source of keratin in nature^2^. Feather waste is a significant source of nitrogen (protein and amino acids); it composed of β-keratins (38%), α (41%) and amorphous (21%) keratin^3–5^. Thus this protein source are attractive and accurate to be used as an alternative to the most favorable organic nitrogen source (peptone & yeast extract etc.), especially in large scale production and it is amusingly to be converted into value added product. Alkali hydrolysis and steam pressure boiling are two common techniques for degrading feathers not only degrade the amino acids but also use a tremendous amount of energy^6,7^. The biodegradation of feathers by microorganisms utilizing keratinase is a practical alternative method or by direct enzymes generated by many microorganisms^8,9^.

Recently, feather waste is considered a new source for different applications like: coating of metals^10^**,** biogas production^11^**,** plant growth^12^**,** leather industry^13^, biofertilizer in agriculture^14^ and keratinase production^15^.

Due to the high cost of a typical complex nitrogen source that are generally used in media preparation such as peptone, yeast extract, casein, etc. feather waste could be an alternative and inexpensive complex nitrogen source for biotechnological manufacturing of some metabolites like: poly-β-hydroxybutyrate^16^, amino acids^17^**,** bio fertilizers^14^ and keratinase^18^. So far there is no previous studies report the usage of feather waste as a binary carbon/nitrogen source for BC production, however many researchers directed to use agricultural, industrial and food processing wastes in BC production as reported recently by El-Gendi et al.^19^. BC is a pure form of cellulose, many of gram positive (G + Ve) and gram negative (G − Ve) bacterial species have been reported as producer of BC. G − Ve bacteria, particularly *Gluconacetobacter xylinus* (*Komagataeibacter xylinus*) is considered a producer model of BC and extensively reported^20^. The major challenge of the biosynthesis process of BC is identifying a new effective, natural and abundant available culture media that can promote a high yield in a short cultivation time and an overall cost reduction. Besides, BC cost-effective production is the prerequisite using wastes as the substrate from locally available wastes such as agriculture wastes; corn stalk^21^, sweet sorghum^22^, tobacco^23^, industrial wastes; waste water of candied jujube^24^, sugar beet molasses^25^, waste fiber sludge^26^ and food processing wastes; starch kitchen wastes, rotten tomatoes, vegetable oil. The benefit of employing these wastes as a medium is not only offers an affordable way to produce BC, but also has an environmentally beneficial impact by removing this waste from the environment and lowering the environmental pollution associated with industrial waste disposal^27^.

On the other hand, food deterioration caused by the infiltration of moisture, oxygen, and, to a lesser extent, heat is one of the main issues in the food sector. The use of polymeric packaging films, such as polyethylene, polystyrene, vinyl polymers and other cellulose-based films, greatly increases the useful lifetime of the food. Economical factors have led to a significant increase in the usage of plastic films in food packaging. Nevertheless, moisture may seep through plastic packaging materials during storage, causing a decline in food quality. After that, moisture adheres to the surface of completely or partially dehydrated foods and seeps inside of them, while oxygen may react with the food’s surface. Both situations eventually result in oxidative deterioration and browning. The physical and transport characteristics of the polymer play a role in how long these processes take. As a result, the choice of polymer for a particular food packaging application has a significant impact on its usable lifetime^28^.

Paper is frequently used as a sustainable packaging material, but it doesn’t have enough water and oil resistance or barrier qualities. The main functions of packaging materials are often to increase oil resistance and avoid or decrease moisture transfer between materials and the surrounding atmosphere. This means the water vapor permeability (WVP) should be as low as possible^29^. Additional coating layers, frequently achieved industrially through lamination with a polymer, are used to assure the protection of paper-based packaging^30^. As chicken feathers (CFs) have distinctive qualities, including lightness, natural abundance and environmental compatibility, keratin is a good raw material for synthetic polymer blends due to its strong mechanical and thermal resistance. The new trend was conducted by applying CFs for coating field development, as it is one of the potential sources of edible coating materials because they are abundant and affordable. Also known as CFs, they are used for coating carbon steel to prevent corrosion^10^. Likewise, it can be used for paper coating to be applied, particularly in food packaging.

CFs signifies a promising candidate as excessively available and cost-effective natural waste from the rapidly growing poultry industry^31^. Numerous studies reported the efficacy of CFs for many heavy metal removals that were attributed to various functional groups in the keratin surfaces^2^, hence increasing the surface area hypothesized to enhance the feather’s absorption capacity. Based upon the above hypothesis, chemical and physical hydrolysis were tried to enhance the metal absorption capacity of the CFs, as the substantial load of heavy metals discharged directly into the water system from several industries represents considerable environmental and economic challenges^32^. Heavy metals are non-biodegradable, persistent and toxic at very low concentrations, resulting in severe health complications by cumulative exposure and consumption^33^. In addition to drinking polluted water, exposure to heavy metals could be indirectly through the consumption of contaminated plants or edible water animals^33^. Besides lead and mercury, contamination with copper, iron, chrome, and cobalt is dominant in most industrial and mining effluents^34^. Cumulative exposure to excessive doses of Cu^++^ is usually associated with serious health complications including hepatic and renal disorders, Alzheimer’s, and lung cancers, where Fe^++^ high doses adversely induce fatal liver and brain damage and/or cancer development^35,36^. Moreover, the adverse effects of Cr^+++^ and Co^++^ exposure on human health are serious and widely reported^37,38^. Several approaches were dedicated to heavy metal removal, including coagulation, metal precipitation and reverse osmosis^32^. However, metal adsorption has currently emerged as a cost-effective, simple and reliable heavy metal approach for wide and commercial applications^37^. Industrial waste implementation in heavy metal removal represents a dual solution for cost reduction and eco-friendly sustainable applications, both economically and environmentally^2,40^.

**Figure 1:**
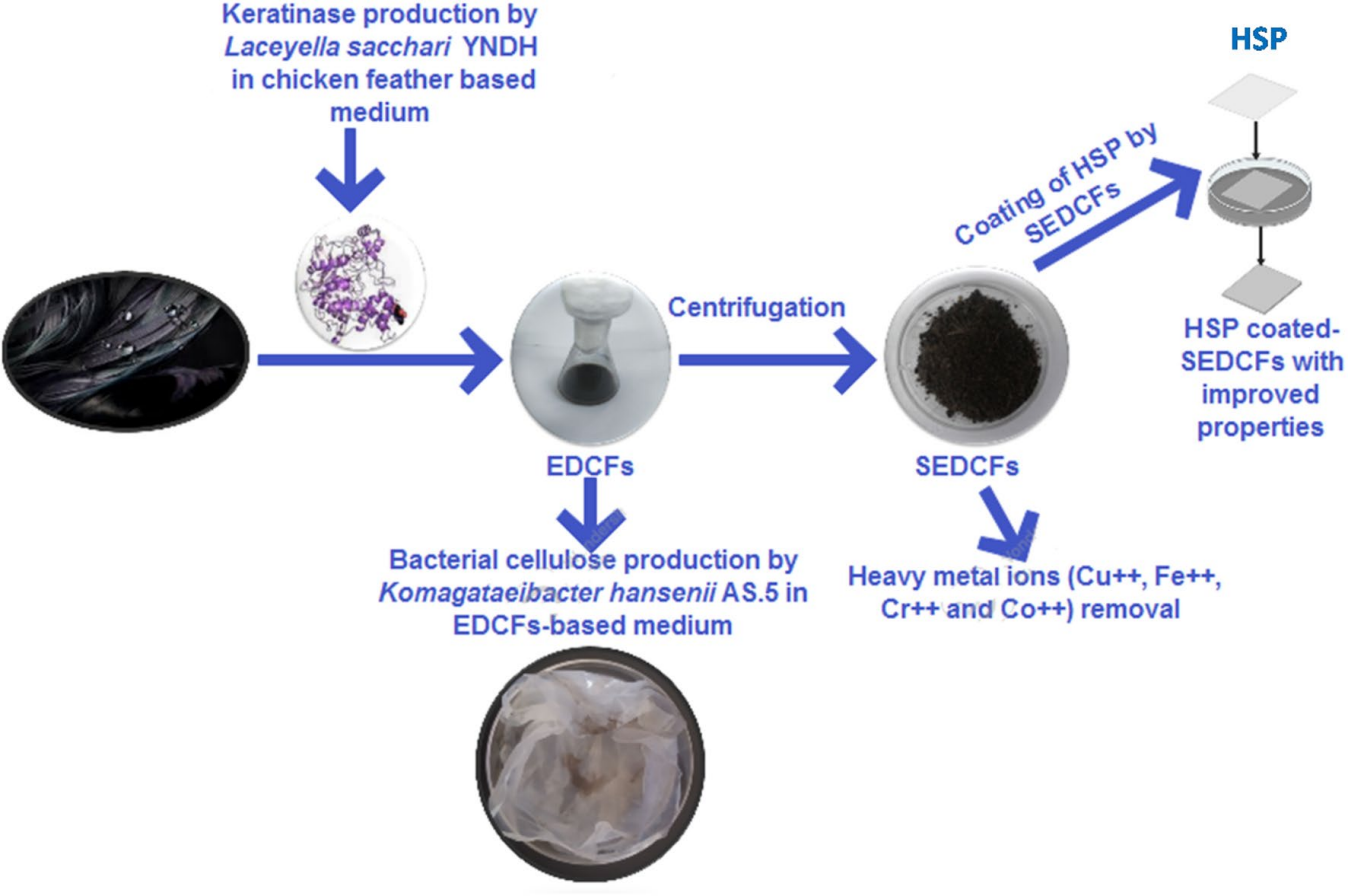
Schematic diagram shows benefiacil multipuropses utilization of bio-hydrolyzed CFs waste liquid lyaste and sludge.

Accordingly, this study provides a cost-effective, promising and beneficial strategy for the usage of both liquid and sludge residues resulting from enzymatically hydrolyzed CFs in green synthesis of cellulose and coating of hand sheet paper (HSP), respectively. Also, the initiation of a successful composite-model for metal ion removal via sludge bio-residue was accomplished. These steps were summarized and entirely illustrated in Fig. 1 using GraphPad Prism 8.0.2 software (https://www.graphpad.com/demos/). To the best of our knowledge, the usage of EDCFs-based medium for BC production and coating of HSP with sludge of EDCFs has not yet been reported in the literature.

## Results and discussion

### Statistical optimization for biocellulose production by multifactorial experiment

Statistical optimization for biocellulose production by multifactorial experiment was carried out following two approaches, Plackett–Burman design (PBD) (the first approach) to determine the relative importance of cultivation variables that affect the generation of biocellulose from *Komagataeibacter hansenii* AS.5 using EDCFs based-medium. The PBD, shown in Table 1, includes nine variables, namely, glucose, yeast extract, peptone, citric acid, pH, time, Na_2_HPO4, temperature and EDCFs. The regression coefficient of glucose, yeast extract, citric acid, pH and time had shown a favorable positive effect on biocellulose biosynthesis, however, peptone, Na_2_HPO_4_, temp and EDCFS had a negative impact. The nine variables were investigated using a linear multiple regression analysis approach and the percent confidence level was derived using the formula: confidence level (percent) = (1− p value)* 100. In addition, the main effect was estimated as the difference between the average measurements of each variable taken at a high (+ 1) and low (1) level. In this experiment, the value of the determination coefficient *R*^2^ was 0.87 for BC production, indicating a high degree of correlation between the experimental and predicted values. The polynomial model describes the relationship between the 9 factors and the biocellulose concentration yield as follows:

$${\text{Y}}_{{\text{cellulose yield}}} \,{ = }\,{0}{\text{.3705}}\,{ + }\,{0}{\text{.0115X}}_{{1}} \,{ + }\,{0}{\text{.01483X}}_{{2}} { - 0}{\text{.0535X}}_{{3}} { - 0}{\text{.0195X}}_{{4}} \,{ + }\,{0}{\text{.0135X}}_{{5}} \,{ + }\,{0}{\text{.08283X}}_{{6}} { - 0}{\text{.198X}}_{{7}} \,{ + }\,{0}{\text{.0138X}}_{{8}} { - 0}{\text{.0345X}}_{{9}} {.}$$

**Table 1:** PB experimental design for evaluating factors influencing on biocellulose concentration produced by *Komagataeibacter hansenii* AS.5 using EDCFs as a carbon and nitrogen source.

Trial	X_1_ (Glucose)	X_2_ (YE)	X_3_ (Peptone)	X_4_ (Na_2_HPO_4_)	X_5_ (Citric acid)	X_6_ (pH)	X_7_ (Temp)	X_8_ (Time)	X_9_ (EDCFs)	Biocellulose conc. (g/L)
1	1 (6)	− 1 (1)	1 (5)	1 (3)	1 (1.5)	− 1 (4)	− 1 (20)	− 1 (6)	1 (200)	0.34
2	− 1 (2)	1 (5)	1 (5)	1 (3)	− 1 (0.5)	− 1 (4)	− 1 (20)	1 (10)	− 1 (100)	0.344
3	1 (6)	1 (5)	− 1 (1)	− 1 (2)	− 1 (0.5)	1 (7)	− 1 (20)	− 1 (6)	1 (200)	0.582
4	− 1 (2)	− 1 (1)	1 (5)	− 1 (2)	− 1 (0.5)	1 (7)	− 1 (20)	1 (10)	1 (200)	0.664
5	− 1 (2)	− 1 (1)	1 (5)	− 1 (2)	1 (1.5)	1 (7)	1 (30)	− 1 (6)	− 1 (100)	0.122
6	− 1 (2)	1 (5)	− 1 (1)	− 1 (2)	1 (1.5)	− 1 (4)	1 (30)	1 (10)	1 (200)	0.124
7	1 (6)	− 1 (1)	− 1 (1)	1 (3)	− 1 (0.5)	1 (7)	1 (30)	1 (10)	− 1 (100)	0.286
8	1 (6)	1 (5)	1 (5)	1 (3)	1 (1.5)	1 (7)	1 (30)	1 (10)	1 (200)	0.236
9	1 (6)	1 (5)	1 (5)	− 1 (2)	− 1 (0.5)	− 1 (4)	1 (30)	− 1 (6)	− 1 (100)	0.196
10	− 1 (2)	1 (5)	− 1 (1)	1 (3)	1 (1.5)	1 (7)	− 1 (20)	− 1 (6)	− 1 (100)	0.83
11	− 1 (2)	− 1 (1)	− 1 (1)	1 (3)	− 1 (0.5)	− 1 (4)	1 (30)	− 1 (6)	1 (200)	0.07
12	− 1 (2)	− 1 (1)	− 1 (1)	− 1 (2)	1 (1.5)	− 1 (4)	− 1 (20)	1 (10)	− 1 (100)	0.652

Levels of independent variables (X1–X9) presented between brackets are expressed in terms of g/L for X1-X5, value for X6-X8 and volume (ml/L) for X9.

− 1 and + 1 constitute the low level and high level and cellulose conc. expressed as g/L.

We employed EDCFs lysate as a medium backbone (carbon and nitrogen source) for BC production since the usage of substituted nutrients greatly lowers the production costs and also diminishes environmental degradation brought on by improperly discarding industrial waste.

The pH, temperature, and cultivation method are physical factors that affect the BC’s microbial productivity. Also, the medium's compositions (carbon, nitrogen and additives) are another critical parameters affecting this bioprocess. Two sequential optimization techniques (PB and BB) are considered popular choices for product optimization^41–44^. Herein, BC production was optimized using the EDCFs as sources of carbon and nitrogen through applying statistical experimental designs. The PB design offers a convenient, quick screening process and quantitatively calculates the significance of a huge number of factors. This saves time and preserves evidence concerning each element^45^. Even if this model does not provide for interaction, the screening programme does not place a high premium on looking into how these numerous elements interact with one another^43^**.**

Of all studied variables, only the most effective and advantageous ones would be selected for further optimization, while those having a significant detrimental impact on the bioprocess might be eliminated from all future tests. The wide range of PB outcomes in this investigation, from 0.34 to 0.65 g/L of BC, highlights the significance of medium tuning for achieving higher productivity.

Analysis of the regression coefficients, *t* test, and *p* values for the nine parameters revealed that glucose, yeast extract, citric acid, pH and time had positive effects on the biosynthesis of BC, but peptone, Na_2_HPO4, temperature, and EDCFs had adverse effects. This is in agreement with Hegde et al.^46^ who found the most effective parameters for BC production were glucose, yeast extract, but is in disagreement with Singh et al.^47^ who reported that pH had a negative effect on bacterial cellulose production.

According to these results, a medium with the following composition (g/L): glucose, 6; yeast extract, 5; Na_2_HPO_4_, 2; citric acid, 1.5; pH, 7; temp, 20 °C; EDCFs waste, 100 ml; time, 10 days, with cellulose concentration, 1.003 g/L, was used as the basic medium for the next design.

**Figure 2:**
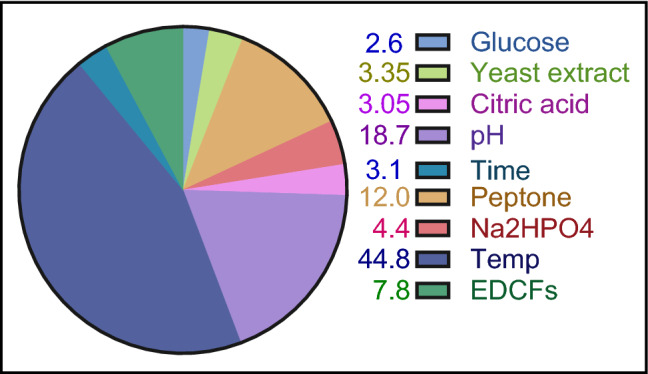
A Pareto chart for PBD that uses EDCFs as a source for carbon and nitrogen to explain the relative importance of each factor in % and its effect on BC production.

Temperature, pH, and EDCFs were chosen because they significantly affect BC synthesis and have a greater confidence level, as shown by a pareto chart (Fig. 2**).** This chart was illustrated by using EdrowMax software (https://www.edrawsoft.com/download-edrawmax.html). In order to simplify the medium and depend mostly on EDCFs lysate as a medium backbone, two verification tests were applied. The first used the typical medium and conditions recommended by the PB equation model, while the second did not include peptone. Both experiments gave approximately the same yield of BC (1 g/L). Therefore, peptone was omitted from the medium in the subsequent experiment.

**Figure 3:**
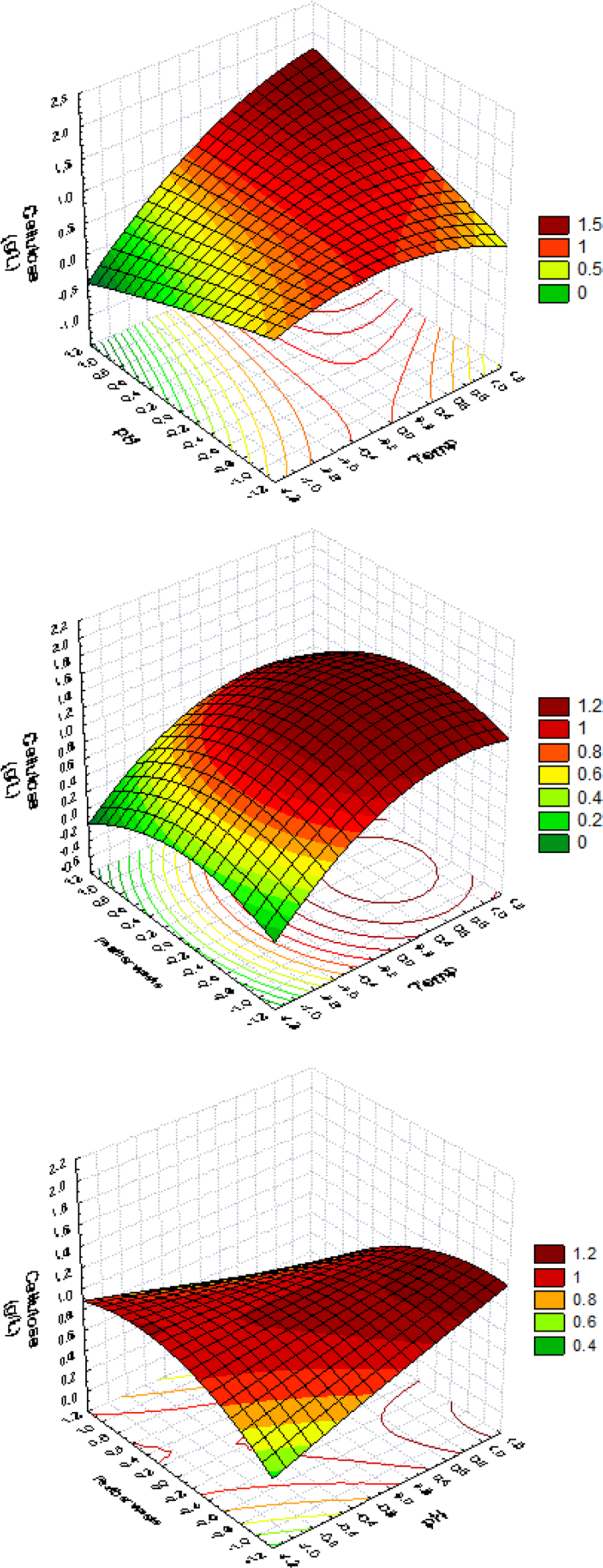
Temperature (X1), pH (X2) and EDCFs (X3) implications on BC production are shown in a 3D response surface and contour graphs.

Accordingly, the major independent variables (X1; temp, X2; pH, X3; EDCFs waste lysate) were further studied at three levels, using Box–Behnken design (BBD) (the second approach) to find the optimum response region for biocellulose synthesis in terms of g/L **(**Table 2**)**. The three variables were studied using a linear multiple regression analysis approach with fourteen trials, and the percentage confidence levels (percent) were calculated as previously described. The determination coefficient *R*^*2*^ = 0.87 for BC concentration, which is a measure of model fit, indicating that only 13% of the responses could not be fitted within the applied model. The use of surface plots to present experimental data demonstrates that higher levels of cellulose production were achieved with lower EDCFs lysate and higher levels of temperatures and pH, as shown in Fig. 3. A second-order polynomial function was fitted to the experimental results for estimating the optimal point of variable within experimental constraints (non-linear optimization algorithm:

$${\text{Y}}_{{\text{cellulose yield}}} \,{ = }\,{1}{\text{.2825}}\,{ + }\,{0}{\text{.4065 X}}_{{1}} \,{ + }\,{0}{\text{.02 X}}_{{2}} { - 0}{\text{.122 X}}_{{3}} \,{ + }\,{0}{\text{.434 X}}_{{1}} {\text{X}}_{{2}} { - 0}{\text{.032 X}}_{{1}} {\text{X}}_{{3}} { - 0}{\text{.24 X}}_{{2}} {\text{X}}_{{3}} { - 0}{\text{.33}}\left( {{\text{X}}_{{1}} } \right)^{{2}} { - 0}{\text{.081}}\left( {{\text{X}}_{{2}} } \right)^{{2}} { - 0}{\text{.272}}\left( {{\text{X}}_{{3}} } \right)^{{2}} {.}$$

**Table 2:** BB experimental design for optimizing the most significant variables influencing on biocellulose concentration produced by *Komagataeibacter hansenii* AS.5 using EDCFs as a carbon and nitrogen source.

Trials	X_1_ (Temp)	X_1_ (Temp)	X_3_ (EDCFs)	X_1_X_2_	X_1_X_3_	X_2_X_3_	X1^2^	X2^2^	X3^2^	Cellulose conc. (g/L)
1	− 1 (13)	− 1 (13)	0 (90)	1	0	0	1	1	0	0.624
2	1 (23)	1 (23)	0 (90)	− 1	0	0	1	1	0	0.602
3	− 1 (13)	− 1 (13)	0 (90)	− 1	0	0	1	1	0	0.266
4	1 (23)	1 (23)	0 (90)	1	0	0	1	1	0	1.98
5	− 1 (13)	− 1 (13)	− 1 (70)	0	1	0	1	0	1	0.356
6	1 (23)	1 (23)	− 1 (70)	0	− 1	0	1	0	1	1.200
7	− 1 (13)	− 1 (13)	1 (110)	0	− 1	0	1	0	1	0.218
8	1 (23)	1 (23)	1 (110)	0	1	0	1	0	1	0.934
9	0 (18)	0 (18)	− 1 (70)	0	0	1	0	1	1	1.04
10	0 (18)	0 (18)	− 1 (70)	0	0	− 1	0	1	1	1.108
11	0 (18)	0 (18)	1 (110)	0	0	− 1	0	1	1	1.246
12	0 (18)	0 (18)	1 (110)	0	0	1	0	1	1	0.324
13	0 (18)	0 (18)	0 (90)	0	0	0	0	0	0	1.281
14	0 (18)	0 (18)	0 (90)	0	0	0	0	0	0	1.284

The optimal levels of the three examined variables, as determined by the polynomial model’s maximum point, were temperature, 23; pH, 8; and EDCFs waste-lysate, 75.26 ml/L, with a predicted cellulose concentration of 1.86 g/L. Finally, to test the quadratic polynomial’s accuracy, a verification experiment was conducted under predicted optimal conditions, measuring biocellulose production in the optimized medium.

The percent accuracy of the model was obtained using the following formula to demonstrate its accuracy: model accuracy = [Y Experiment/Y Calculated] 100. The Y value is 2 g/L, according to bench scale studies. The accuracy of the calculated model was 107.5%. In this work, a statistical methodology based on a combination of PB and BB designs was found to be successful and accurate in identifying statistically important components and determining their optimum concentrations. As a result, the following medium composition (g/L) is estimated to be close to the optimum: glucose, 6; yeast extract, 5; Na_2_HPO_4_, 2; citric acid, 1.5; pH, 8; temperature, 23 °C; EDCFs waste, 75.26 ml; and time, 10 days under static condition, where BC concentration was 2 g/L and the yield increased twofold compared with production from the PB design.

Experimental data presented as surface plots reveals that high BC production yield is supported by higher temperatures and pH levels, even at relatively low EDCFs lysate waste levels.

For BC production, the *R*^*2*^ value was 0.87, indicating a strong association between experimental and forecast values. Experimentally, the confirmed optimal conditions from the optimization experiment were compared to the model’s anticipated value. The estimated BC concentration was 2.1 g/L, and the predicted value from the polynomial model was 1.87 g/L. This high level of accuracy (107.5%) shows that the model was validated under ideal circumstances; furthermore, the BC concentration determined by BBD was 2 times higher than PBD. This demonstrates the significance and necessity of the optimization process. Our research supports the findings of^46,48^, they claim that the RSM is a generally accepted modern statistical strategy for the optimization of the experiment's overall circumstances and the solution of the analysis problems. RSM assists in identifying the critical variables for studying interactions, determining the optimal number of variables, and ensuring the best output in a limited number of tests^49^. In conclusion, employing EDCFs for BC manufacture can save production costs and environmental pollution.

### Statistical analysis

“JMPIN Version: 4.0.4 software was used to perform the experimental designs and statistical analysis and STATISTICA software version 7 was used to draw 3D surface plots”.

### Chemical structure characterization of BC membranes

#### Fourier transform infrared (FTIR) spectroscopy-analysis

**Figure 4:**
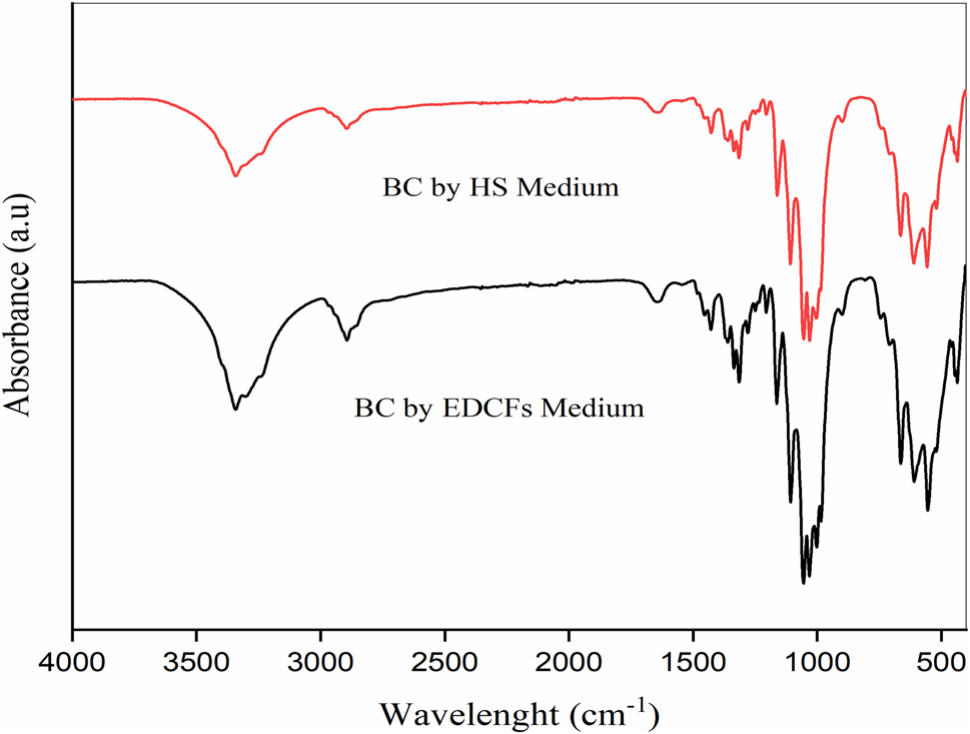
FTIR spectra of BC by HS medium (red) and that based on EDCFs medium (black) lines.

The chemical structure of the BC membrane based on EDCFs medium was compared with the standard HS medium, where FTIR spectra of both BC membranes are shown in Fig. 4. Basically, no differences in characteristic peaks of BC in either HS medium or based on EDCFs medium were observed^50^. It was observed that the absorption band assigned to the –OH groups of cellulose appears at ν 3350 cm^−1^. Other cellulose-specific peaks were found at ν 2894, 1427, 1350, 870 and 655 cm^−1^ which are assigned to –CH stretching bands, (HCH, OCH) bending inside of plane vibration, –CH deformation vibration, (COC, CCO, CCH) deformation mode stretching vibrations, and C–OH banding out of plane; respectively^50,51^.

#### X-ray diffraction (XRD) analysis

**Figure 5:**
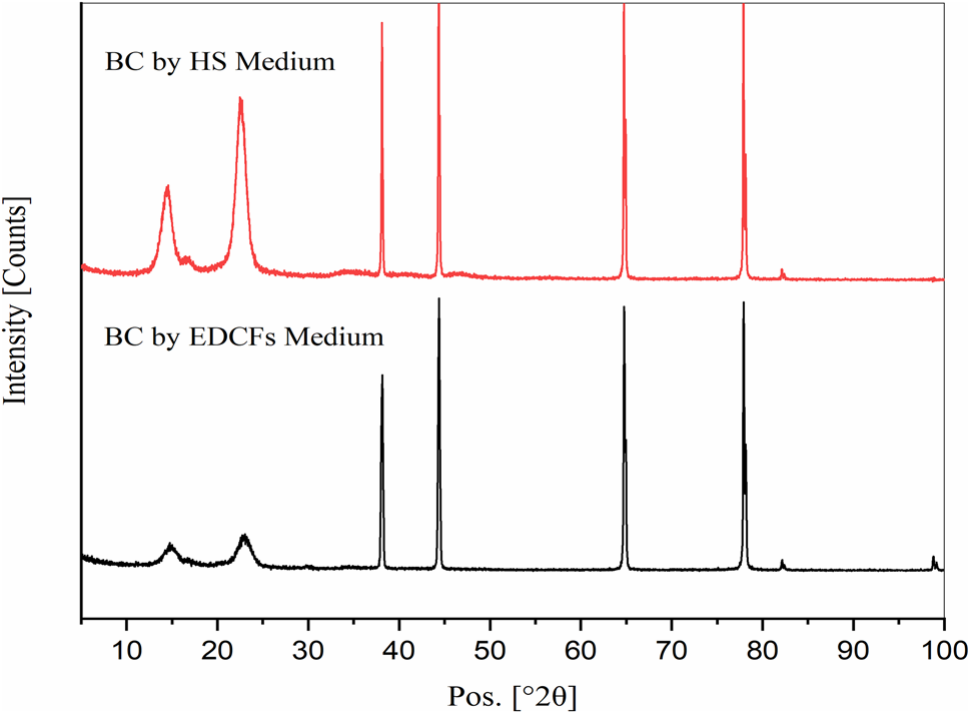
XRD patterns of BC membranes by HS medium (red) and by EDCFs medium (black).

Figure 5 illustrates the XRD diffraction patterns of both two BC obtained by HS medium and by EDCFs optimized medium. As seen in Fig. 5, no differences in the characteristic peak patterns of two BC membranes are observed. The diffraction diagrams of BC reveal more than two characteristic diffraction peaks, indicating that BC contains *I*_*α*_ and *I*_*β*_ crystal cellulose. Generally, two BC membranes show a high degree of crystallinity or crystallinity-index between 90–95%, where two BC membranes reveal the characteristic diffraction peaks at 2θ = 16° and 25° with interplaner spacing (*d-spacing*) 3.91 and 2.32, respectively^50^. Meanwhile, other diffraction peaks are observed at 2θ = 38°, 45°, 64° and 78° which indicate the presence of unreacted or unconverted glucose or amino acids residues of BC obtained from HS medium or produced BC from EDCFs medium, respectively^50,52^.

#### Scanning electron microscopy (SEM) investigation

**Figure 6:**
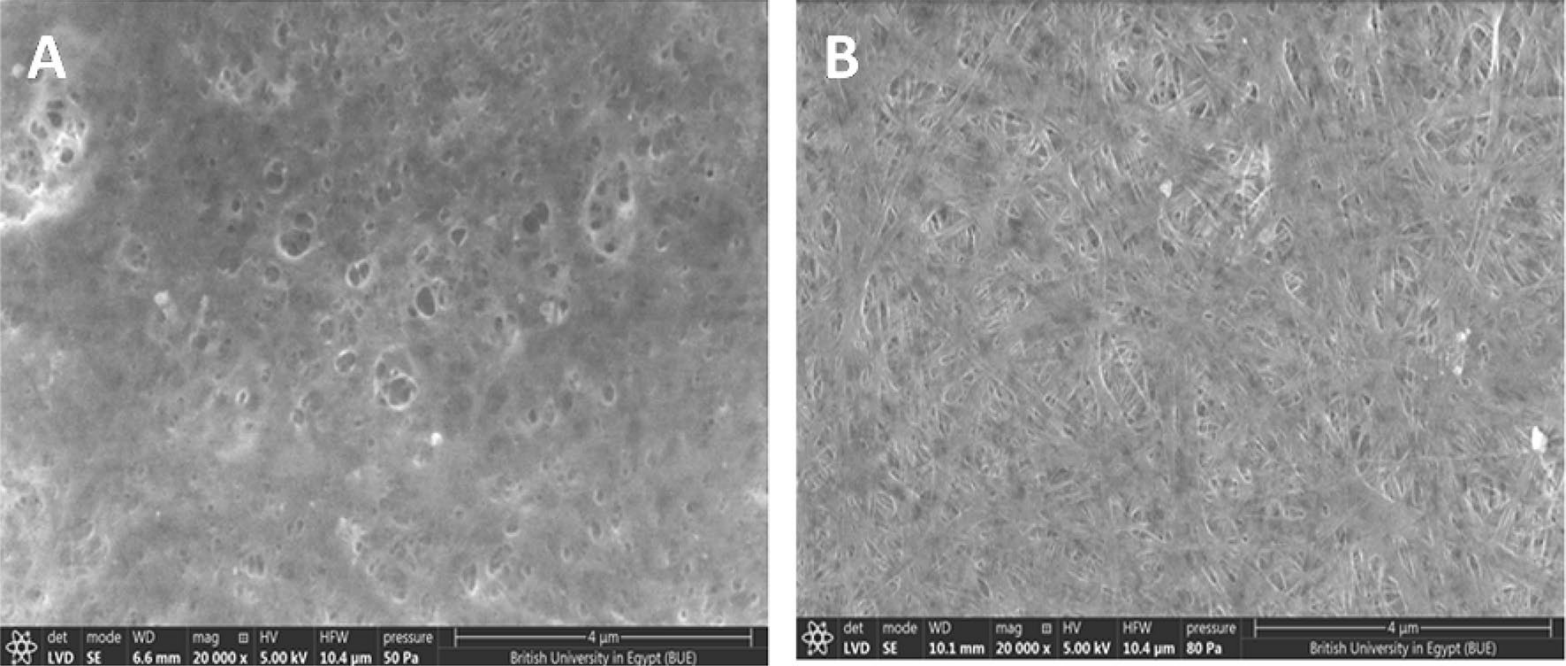
SEM image of BC obtained from HS medium (**A**) and from EDCFs based medium (**B**) at magnification power 20,000X.

The morphological properties of BC membranes, which were produced by both HS and EDCFs medium, were examined by SEM with different original magnifications at 10,000 and 20,000X, as shown in Fig. 6. As seen, the surface morphology of BC membrane based on EDCFs seems to be more uniform, with a filamentous shape structure, ribbon-fibril networks, and pours less, particularly with high magnification, compared to traditional bacterial cellulose produced by HS medium (Fig. 6). Notable, fibers of BC produced by EDCFs medium diameters were found to be an average of 50–100 nm, whereas diameters of bacterial cellulose produced by HS medium were found to be between 100–150 nm. The current SEM investigation is almost consistent with SEM investigations as previously reported by Tsouko et al.^53^ and Zhang et al.^54^.

### Characterization of SEDCFs

#### FT-IR analysis

**Figure 7:**
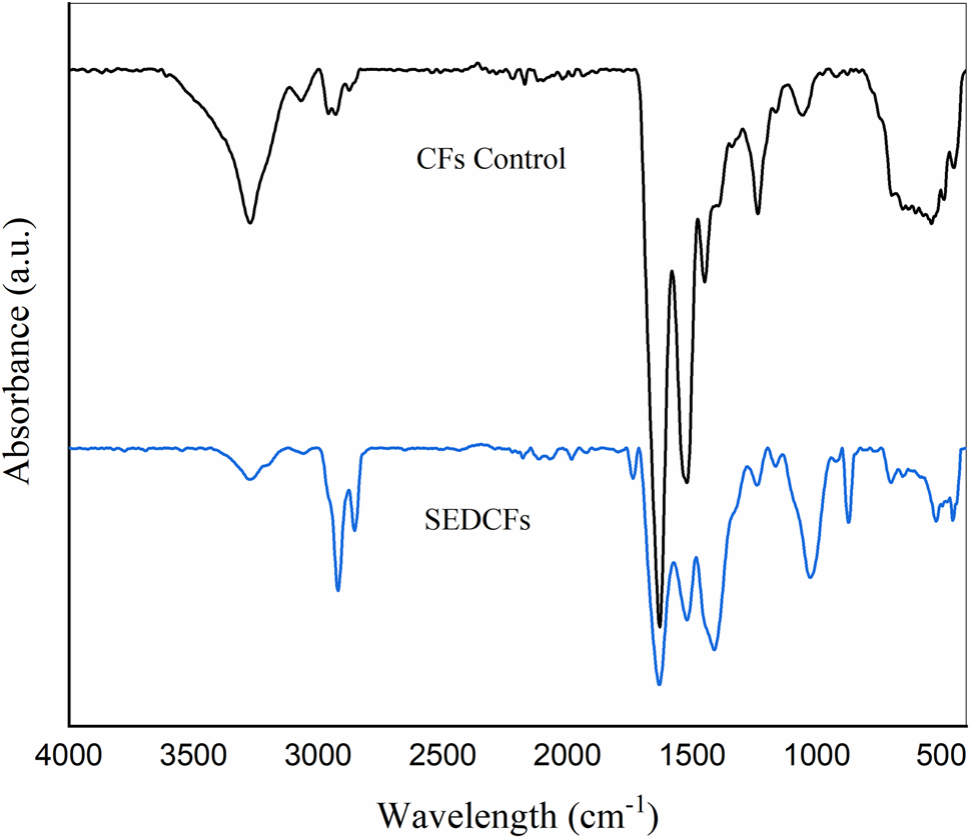
FT-IR spectra of CFs control (black) and SEDCFs (blue).

For understanding the chemical structure of pristine CFs and SEDCFs, FT-IR spectra were conducted (Fig. 7). After comparison of control CFs and treated SEDCFs samples, it was observed that the characteristic peaks are mostly similar to each other and are comparable with each other. On the other hand, the chemical structure of SEDCFs exhibits little effect on the chemical structure of protein after degradation (Fig. 7, right). The transmission band region between ν 3500–3200 cm^−1^ in CFs, was shifted to ν 3500–3000 cm^−1^ in SEDCFs due to starching vibration of –O–H and –N–H of amide A. However, bands that appeared in the range between ν 3000–2800 cm^−1^ were related to symmetrical –CH_3_ stretching vibration in treated SEDCFs, only^52,53^. Interestingly, in case treated SEDCFs; strong absorbance band at 1730–1630 cm^−1^ which is attributed to C=O stretching of amide I. Also, the absorption peak at ν 1520–1410 cm^−1^ is attributed to N–H bending and C–H starching, of amide II of SEDCFs. While, weak band at ν 1240 cm^−1^ is associated with amide III derived from N–H bending, C–N stretching and some bending from C=O bending and C–C stretching vibration of SEDCFs. Also, a weak peak at ν 700 cm^−1^ is related to the N–H out-of-plan bending of treated SEDCFs^53^. Notably, strong vibration peak around ν 1730 cm^−1^ is attributed to C=O of fatty acid ester found in animal skins, and was detected only in cases of treated SEDCFs, which confirms that the treatment of degradation does not affect the main structure of keratin^55^. However, the C–O stretching vibration associated with ester linkage, attributed at around 1230 cm^−1^ was detected in both CFs control and treated SEDCFs^50,53,55^. Overall, it was shown that there are no effects on the main chemical composition of CFs after degradation treatment, but the chemical composition of SEDCFs becomes more clear and precise.

#### Wide-angle X-ray diffraction (WAXRD)

**Figure 8:**
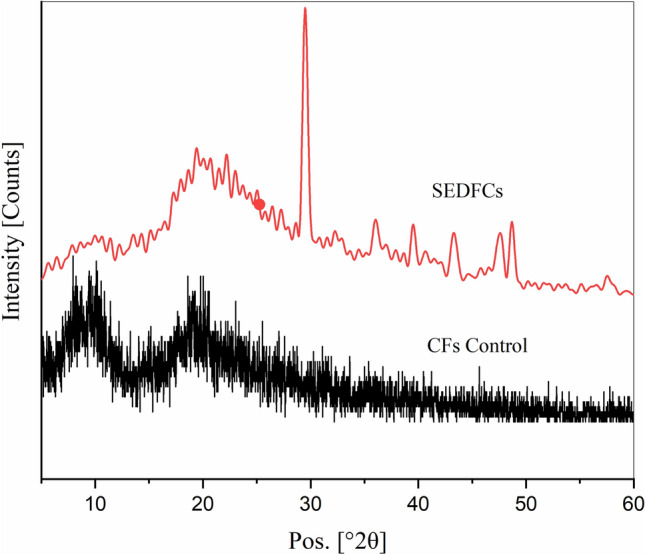
XRD patterns of CFs control (black) and SEDCFs (red).

WAXRD was used to determine the crystal phase of the tested CFs control and SEDCFs samples (Fig. 8). The XRD patterns in Fig. 8 show that both CFs and SEDCFs mainly existed in the semi-crystalline phase, and even after some hydrolysis, they retained the crystallinity. As seen, CFs show diffraction characteristics of α-helix appearing at 2θ = 9.5° and β-sheet at 2θ = 20.8°, however SEDCFs exhibit diffraction characteristics of shifted α-helix appearing at 2θ = 10.6° and β-sheet at 2θ = 21.8°^50,53,55^. While, the diffraction peaks at 2θ = 13° and 16° of CFs and SEDCFs, respectively are allocated for the amorphous region. Also, diffraction peaks are allocated at 2θ = 29° and 38° were indexed for theβ-sheet crystalline structure of SEDCFs, while the peaks between 2θ = 17°-20° indexed for α-helix diffraction patterns of SEDCFs. Overall, XRD results indicate that partial crystallinity of SEDCFs is retained after the enzymatic degradation process, compared to pristine CFs.

#### Morphological investigation

**Figure 9:**
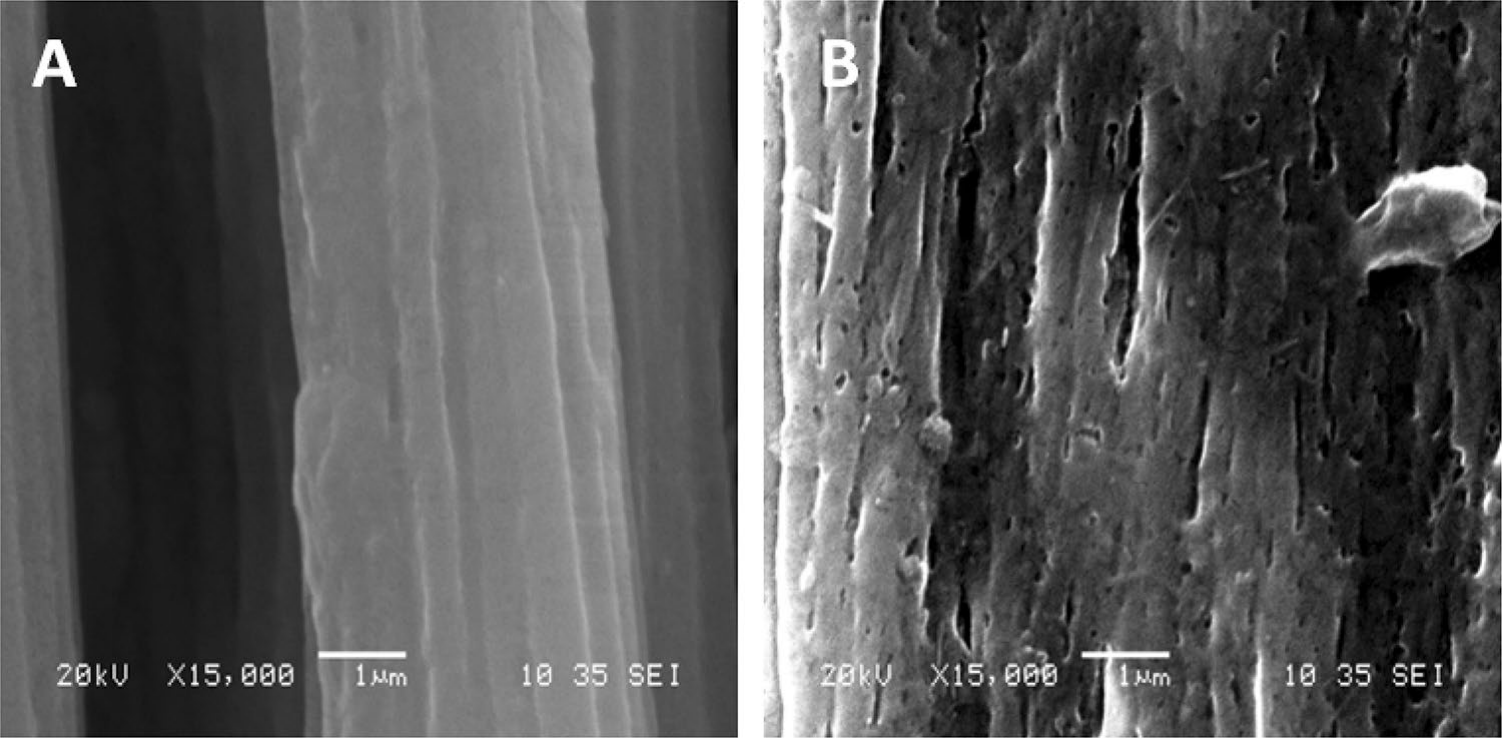
SEM photographs of CF_S_ (**A**) and SEDCF_S_ (**B**) at 15000X magnification power and 20 kV.

The conclusion of untreated and treated CFs morphology was examined via necked eye and using SEM investigation. The outcomes exhibited the presence of all main components of CFs even after degradation treatment with minimum differentiation. However, further morphological investigation and elemental analyses were conducted by SEM and SEM–EDX analyses, as shown in Fig. 9, treated and degraded SEDCFs images were displayed with different magnifications in Fig. 9. As seen, SEDCFs were kept somewhat erection even after degradation treatment as found in pristine CFs, with a lack of woolly-shape structure parts as compared with control CFs (Fig. 9).

#### Mechanical properties for coated HSP

The use of CFs extract as an ecofriendly method for the development of composites and coating agents for the protection of carbon steel from corrosion^10^. This is the first report to use sludge of enzymatically degraded chicken feathers (SEDCFs) as a coating agent for HSP. The HSP was coated to determine its mechanical performance using three different concentrations of SEDCFs (1, 2 and 3%). Table 3 shows the results of mechanical properties, as bulk densities, maximum load, breaking length, tensile index, Young’s modulus, work to break, and coating layer. Bulk densities, described as the reciprocal value of density that specifies the compactness or volume of the paper, can be used to conclude the relationship between both the grammage and thickness of paper^56^. The bulk density of blank HSP was more significant than that reported from coated HSP, indicating the opposite the values of the coated HSPs, bulk density shows a reduction upon increasing the content of SEDCFs, thus suggesting that the presence of SEDCFs may affect the cellulosic fibers of HSP, this is compatible with other study^57^. Table 3 shows that with increasing the concentration of SEDCFs, the maximum load and breaking length were increased; this was due to the strong coated HSP formed by the action of coating agent. This result is in agreement with other work^58^. The tensile index is (tensile strength/base weight) a mechanical variable that characterizes tensile strength in relation to material quantity. This variable is influenced by the degree of fiber bond formation^59^. Results represented in Table 3 indicate that the low value of the tensile index was observed in the HSP coated with SEDCFs when compared with blank HSP, so that the tensile index exhibited negligible changes in mechanical properties, this may be due to the large penetration of the coating solutions into the HSP at high coating ratio led to the swelling of the cellulose fibers, which further decreased their mechanical properties^60^**.**This results are consistent with other research^61^.Young’s modulus result of coated HSP refers to the fact that there is no significance was observed due to the action of coated SEDCFs against to blank HSP. The resulting SEDCFs -coated HSP showed increased work at break, this result indicated the flexibility of the coated feather over the HSP, this observation are in agreement with^62^, who coated the HSP with cellulose stearoyl ester, on the other hand, other study reported that with increasing the concentration of coating agent (cellulose nanofiber/chitosan nanoparticles), the value of work at break decreased^63^. In general, a positive effect on the mechanical behavior of coated HSP is induced by the presence of SEDCFs with a relevant increase on both maximum load and work at break.

**Table 3:** Mechanical properties of HSP coated with SEDCFs (bulk densities, maximum load, breaking length, tensile index, Young’s modulus, work to break and coating layer).

Feather concentration	Bulk density (g/m^3^)		Maximum load (N)		Breaking length (km)		Tensile index (N.m/g)	
		% improvement		% improvement		% improvement		% improvement
Blank	884.96		17.00		3.31		460.80	
1 g	639.13	27.78	20.72	21.92	2.45	25.92	377.53	18.07
2 g	601.83	31.99	21.98	29.30	2.60	21.23	418.94	9.08
3 g	563.94	36.27	23.72	39.54	2.80	15.21	386.68	16.08

	Young’s modulus (MPa)		Work to break (J)		coating layer (g/m^2^)			
		% Improvement		% Improvement		% Improvement		
Blank	6803.63		0.01			0.01		
1 g	2412.95	64.53	0.02	45.55	4.12	0.02		
2 g	2521.34	62.94	0.02	71.74	4.72	0.02		
3 g	2180.66	67.95	0.03	94.31	5.90	0.03		

#### Water vapor permeability (WVP)

**Figure 10:**
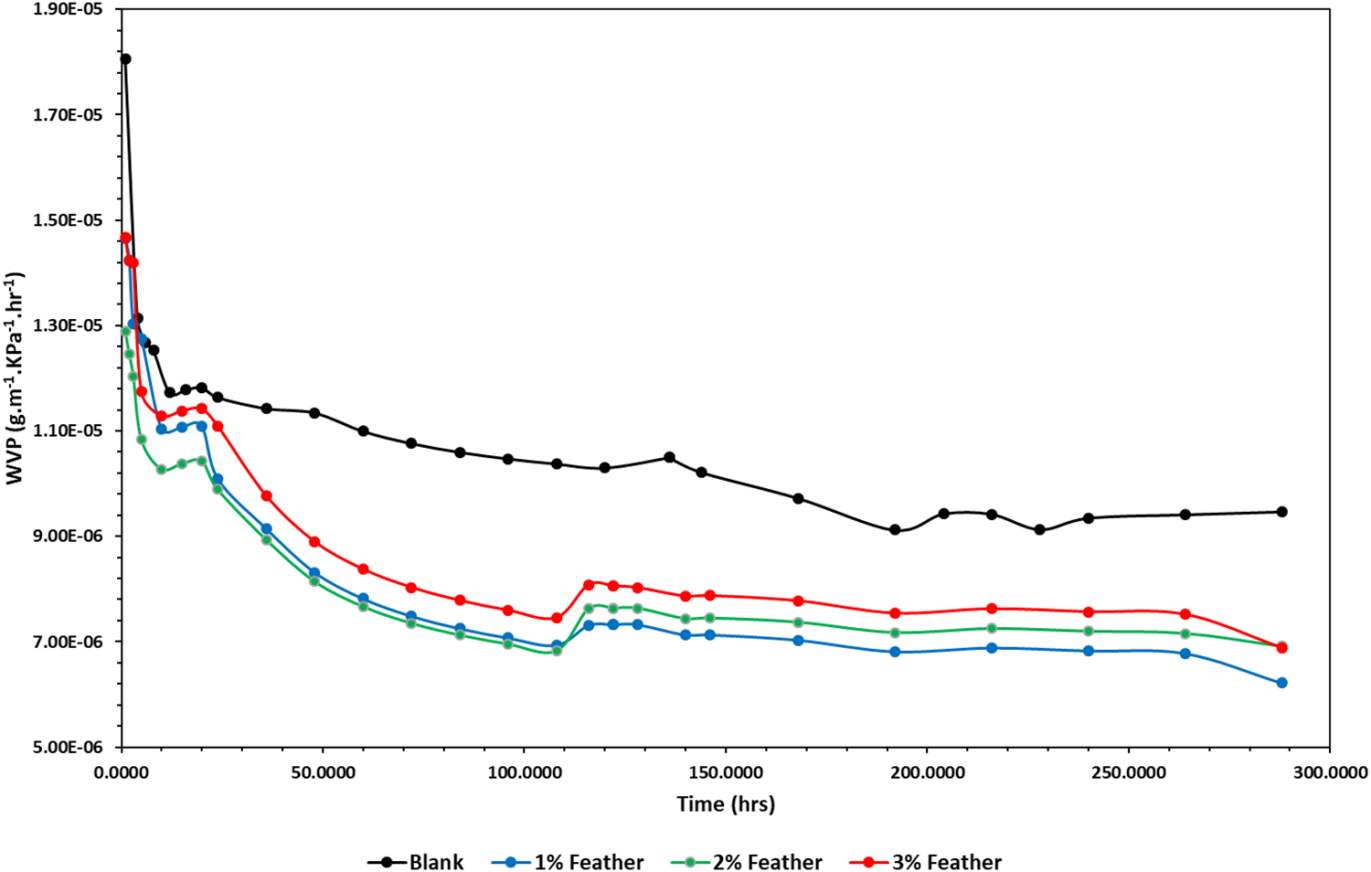
The WVP of none coated HSP (blank) and coated HSP with SEDCFs at different concentrations of SEDCFs (1%, 2% and 3%).

The WVP of HSP was defined as the mass of water vapor passing through the HSP per unit area and time under defined conditions. The low WVP values are desirable for industrial packaging like food, drugs, and instruments^62^.WVP of the coated HSP was estimated as well and compared with the uncoated HSP as presented in Fig. 10. It is evident that coated HSP exhibits higher resistance to WVP compared to uncoated HSP. Although SEDCFs have a strong hydrophilic performance, the usage of SEDCFs in paper coating results in lower water vapor penetration through the coated HSP and consequently improves hydrophobicity. In particular, the efficiency of the SEDCFs in reducing the WVP is evident even at low concentration (1%), reaching an improvement in WVP up to 18%. This could be due to the low concentration of feather extract blocking the pour size of HSP.On the other hand, as the concentrations of SEDCFs increased the WVP of coated HSP increased. This explained by the aggregation and coagulation of SEDCFs at high concentration on the surface of HSP. These findings are consistent with previous research, which found that a low concentration of sodium alginate/nanocellulose/Ag-NPs nanocomposite decreased the WVP of coated, whereas a higher concentration of cellulose nanocrystal/Ag-NPs increased the WVP of coated paper^64^.

#### Oil resistant barrier (ORB)

The ORB of the coated HSP was investigated and compared with the HSP as illustrated in Table 4. The results showed that HSP coated by SEDCFs had remarkable oil resistance when olive oil was evaluated. Herein, HSP and coated HSP were tested for their oil-resistance using the olive oil assay, where the time required for penetration of olive oil through the sample is observed. The results of the test showed that coated HSP at different concentrations from SEDCFs showed excellent grease proof property since the time needed for penetration of olive oil was > 600 s, which can be classified as high grease-proof materials. As well as, with the SEDCFs concentration increase, the oil resistance of HSP increased. The coated HSP exhibited a good ORB (130–600 s) compared with the uncoated HSP (80 s). Coatings based on SEDCFs are highly lipophilic materials, such that hygroscopic oil does not dissolve in its HSP, leading to excellent ORB^65^. The oil resistance of paper coated with different SEDCFs concentration exhibited good oil resistance as the SEDCFs concentration increased from (1–3%) due to the high hydrophobicity of it, which meets the demands for food packaging. Several studies were reported, the addition of active materials as coated part enhance their ORB as cellulose nanofibers/chitosan nanoparticles^65^, HSP/ZnO/SiO2^58^ and HSP/Microcrystalline Wax Emulsion^66^.

**Table 4:** Resistance of uncoated and coated HSP with EDCFs for olive oil penetration.

Sample reference	Average time (s) (Time needed for penetration of olive oil)	Resistance %
Blank	80 ± 10	100
1%	130 ± 9	162.5
2%	140 ± 10	175
3%	600 ± 15	750

#### Removal of heavy metals using SEDCFs

**Figure 11:**
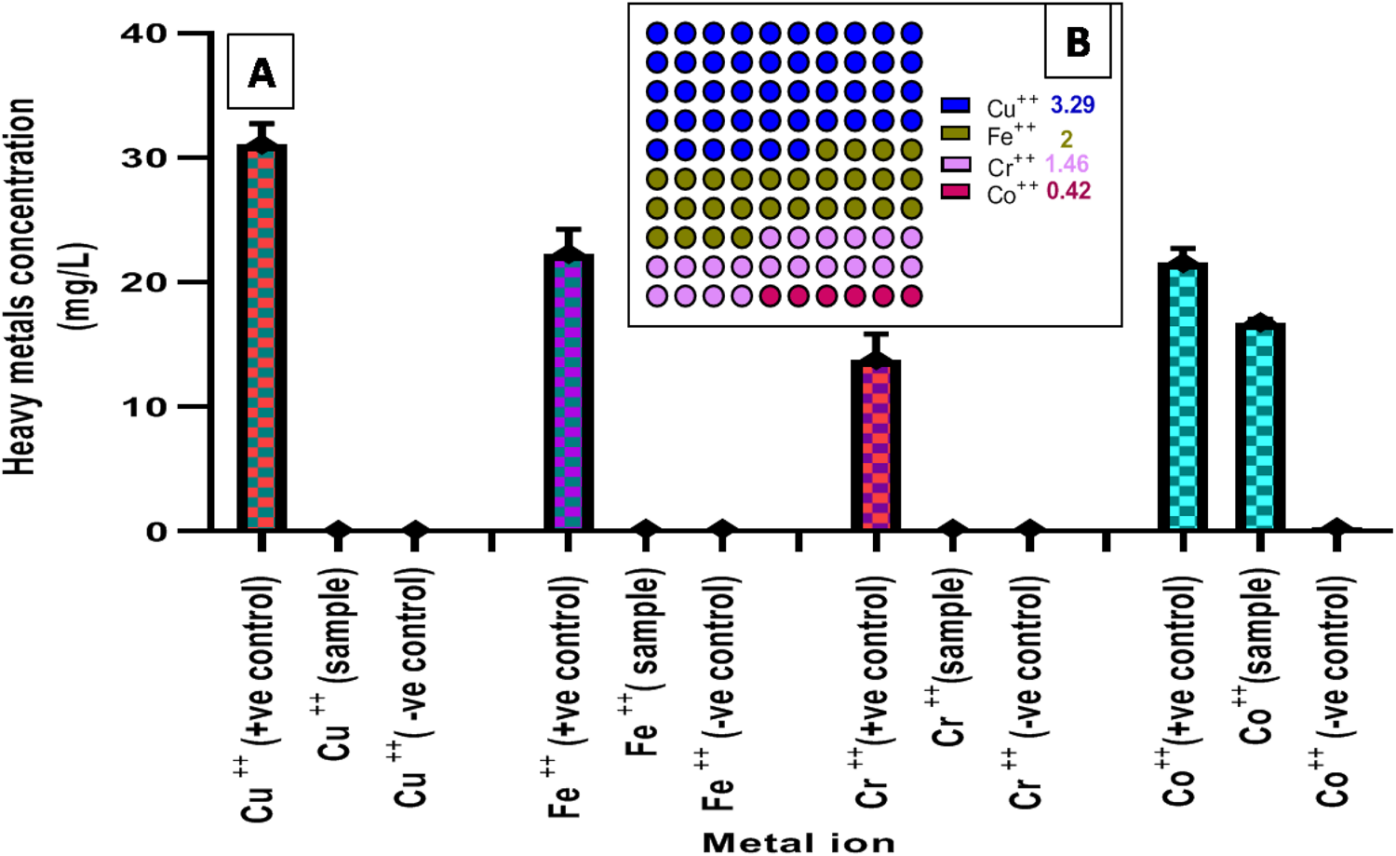
The affinity of SEDCFs toward Cu^++^, Fe^++^, Cr^++^ and Co^++^ in metal solutions (**A**) and its sorption capacity q (mg/g) (**B**).

Heavy metals accumulation represents a great environmental challenge attributed to the adverse effects on human and animal health^33^. Several approaches are currently reported for heavy metal removal. However, the cost implemented, energy consumption, and impact of the environmental impacts of the metal removal method are still challenging^31^. On the other hand, CF is a main waste in the poultry industry with high environmental impacts^31^. In this regard, the efficiency of the SEDCFs on heavy metal removal was evaluated toward four model metal ions, namely, Cu^++^, Fe^++^, Cr^++^ and Co^++^. The results **(**Fig. 11) indicate a significant affinity of SEDCFs toward Cu^++^, Fe^++^ and Cr^++^,which were completely removed from metal solutions **(**Fig. 11). The SEDCFs affinity ability toward the three metals was in the following order: Cu > Fe > Cr as indicated in the sorption capacity results: 3.29, 2.017 and 1.46 mg/g for the three metals, respectively. The high potency of feather waste for Cr^++^ and Cu^++^ has been reported in other studies^67^.

In the same regard, the metal removal was reported for CF in Pb^++^ removal from wastewater by de la Rosa et al.^68^, and also for CFs-ash for Cd^++^ removal from waste water^39^. On the other hand, the SEDCFs revealed a low affinity toward cobalt ions, revealing only 18% Co^++^ removal from aqueous solution with a sorption capacity of about 0.42 mg/g. The results are in accordant with Zhang et al.^67^ who reported the low affinity of CFs in addition to three other natural keratin sources, toward cobalt ions, and contrary to Chakraborty et al.^35^, reporting the opposite. The low affinity toward cobalt ions, in the current study, asserts the selectivity of biologically prepared SEDCFs toward different metals that could be attributed to the diverse functional groups on the SEDCFs sludge particles’ surfaces^2,67^. As per literature, the CF was hypothesized to remediate heavy metals through surface precipitation in addition to ion exchange between the CF surface’s Ca^++^ ions and the targeted heavy metal^39^.

## Conclusion

This study highlights the production of biocellulose by *Komagataeibacter hansenii* AS by using a new alternative carbon/nitrogen source namely, CFs lysate in submerged fermentation. A complete green sustainable bioprocess was applied successfully and the optimal conditions were attained. The results indicate that the EDCFs waste product macromolecules could be used as a good nutritional ingredient of culture media not only for BC but for any other metabolites; this is implying a potential economic and environmental benefits. Several properties and structural morphology of BC produced from EDCFs waste by- products were compared to that from synthetic medium, where they are compatible. On the other hand the developed sludge upon enzymatic hydrolysis of CFs (SEDCFs) was applied for coating HSP as a green composite. The newly developed bio-composite showed an improvement in paper flexibility, less water permeability and remarkable oil resistance compared to uncoated paper. Additionally, the efficiency of the SEDCFs on heavy metal ions removal was evaluated toward four model metal ions namely, Cu^++^, Fe^++^, Cr^++^ and Co^++^. A significant high affinity of SEDCFs was recorded toward Cu^++^, Fe^++^, Cr^++^ and low affinity towards Co^++^ in metal solution.

## Material and methods

### Gathering and processing of feathers

CFs was purchased from a nearby market. Feathers were washed with detergent and then washed again with tap water to remove the detergent. Waste feathers were washed and dried at 50 °C for 6 h before being subjected to enzymatic breakdown^67^.

### Inoculum preparation

Cells of *Laceyella sacchari* YNDH from a freshly produced plate (starch/nitrate-agar medium) were allowed to grow in a 50 ml aliquot of starch/nitrate broth medium that was dispensed in a 250 ml Erlenmeyer flask as the inoculum. The incubation process was conducted for roughly 48 h at 45 °C and 200 rpm^69^.

### Preparation and manufacturing of protease/keratinase

Protease/keratinase enzyme produced by *Laceyella sacchari* YNDH was generated in the optimized production medium^67^, after 48 h fermentation period; the broth was collected, centrifuged for 20 min, and the supernatant filtered using 0.2-filters (MDI, India) using a vacuum pump (WATSON-MARLOW-101 U/R).

### Enzymatically degradable chicken feathers (EDCFs) and sludge of enzymatically degradable chicken feathers (SEDCFs) preparations.

As previously reported by Doaa et al.^70^, the micro filtered supernatant was employed as the crude enzyme for feather breakdown and feather meal synthesis by adding it directly to the feather wastes. Feather lysate in total included both liquid and sludge has been used in biosynthesis of bacterial cellulose while, for sludge separation, this lysate has been centrifuged for 20 min at 5000 rpm, washed with 70% ethanol followed by distilled water and dried for 8 h at 40 °C for further applications in heavy metal removal and HSP coating.

### Biocellulose production

#### Microorganism

An Egyptian local isolate has been previously identified as *Komagataeibacter hansenii* AS^20^, deposited in GenBank at accession number MH109871 and known as a producer of biocellulose was applied in this study. The culture (slant form) was kept at 4 °C using Hestrin and Schramm medium (HS) agar for short term strain preservation. The HS broth medium composed of (g/L): 20 D-glucose, 5 peptone, 5 yeast extract, 2.7 disodium hydrogen phosphate, 1.15 citric acid and ethanol 5 ml (pH 5.5) was used for preinoclume preparation upon incubation the inoculated broth medium under shaking at 30 °C for 2 days for culture activation as reported by Saleh et al.^20^.

#### Statistical optimization for production of biocellulose using EDCFs

In two steps, physicochemical variables for the synthesis of biocellulose from *Komagataeibacter hansenii* AS5 using EDCFs as carbon/ nitrogen sources were optimized. The first was the use of PBD to filter physicochemical factors. The second was to use BBD to optimize the most important factors that influence the synthesis of biocellulose processes depending on EDCFs a medium backbone.

#### PBD

The design was utilized to identify the important factors that had a substantial impact on the amount of biocellulose produced when EDCFs debris was employed among medium components. By using EDCFs waste as a carbon/nitrogn source, a PB experimental design with a set of 12 experiments (trials) was utilized to determine the relative significance of nine factors or variables (glucose, yeast extract, peptone, NaHPO_4_, citric acid, pH, temp. time & EDCFs) that influenced biocellulose formation. PBD is based on the first-order model Y = β_0_ + ∑ β_i_ x_i_, where in this model, the response is represented by Y, the model intercept is represented by β_0_, the variable estimate is represented by βi and the variable is represented by xi. The *p* value was calculated using standard regression analysis to determine the weight of the studied variables. Table 1 shows the investigated factors, as well as the values of each component in the experimental design and the measured response (cellulose conc. g/L). A high (+ 1) and low (− 1) concentration was evaluated for each component.

All trials were done in triplicate (using 50 mL medium in 250 mL Erlenmeyer flasks), and the average value for the measured response was computed. Using BBD, response surface methodology (RSM) was employed to optimize the screened components for better biocellulose production. After calculating the relative significance of independent variables, the three major significant factors were chosen for further evaluation in terms of cellulose conc. (g/L) as a response after 10 days of incubation time under static condition.

#### BBD

BBD was applied for optimizing the production of biocellulose using EDCFs lysate fraction as carbon and nitrogen sources, where the conducting statistically planned trials, estimating the coefficients of the constructed mathematical model, anticipating the response, and judging the model's appropriateness were all part of the optimization approach^71^. Table 2 shows the design matrix (containing 12 trials), three levels (high, medium and low) for the chosen variables (denoted by + 1, 0, and − 1) and 2 central trials to find faults in handling as well as the measured response (biocellulose conc.)^72^. The coefficient results of each variable were used to apply the model^73^. The following second-order polynomial structured model was used to predict biocellulose conc. (Y) as a function of cultivation conditions (X) for three variables:

$${\text{Y}}\,{ = }\,{\upbeta }_{0} \,{ + }\,{\upbeta }_{1} {\text{X}}_{{1}} \,{ + }\,{\upbeta }_{2} {\text{X}}_{{2}} \,{ + }\,{\upbeta }_{3} {\text{X}}_{{3}} \,{ + }\,{\upbeta }_{12} {\text{X}}_{{1}} {\text{X}}_{{2}} \,{ + }\,{\upbeta }_{13} {\text{X}}_{{1}} {\text{X}}_{{3}} \,{ + }\,{\upbeta }_{23} {\text{X}}_{{2}} {\text{X}}_{{3}} \,{ + }\,{\upbeta }_{11} {\text{(X}}_{{1}} {)}^{{2}} \,{ + }\,{\upbeta }_{22} {\text{(X}}_{{2}} {)}^{{2}} \,{ + }\,{\upbeta }_{33} {\text{(X}}_{{3}} {)}^{{2}} {.}$$

Y is the anticipated response; β_0_ is the model intercept; X_1_, X_2_ and X_3_ are the independent variables; β_1_, β_2_ and β_3_ are linear coefficients; β_12_, β_13_ and β_23_ are cross product coefficient s; and β_11_, β_22_ and β_33_ are the quadratic coefficient.

### Quantification of BC concentration/yield gravimetrically

At the end of the cultivation time, the observed BC pellicle at the air–liquid interface was collected and washed several times with distilled water to get rid of the excess medium components. Afterwards, the BC sample was then soacked three times in 0.5% sodium hydroxide at 90 °C for 30 min, to remove bacterial contaminants and other impurities immobilized on the BC, and then washed with distilled water until neutralization. Finally, the purified BC sample was dried in the oven at 50 °C until a constant weight was recorded^74^.

### Data analysis using statistical techniques

Multiple linear regressions were used to estimate the *t *values, *p* values, and confidence levels for biocellulose production yield using data analysis JMP software. The Student *t* test was used to evaluate the significance level (*p* value). The *t* test for any individual effect allows for an assessment of the likelihood of discovering the observed effect by chance. It will be accepted if the probability of the variable under test is low enough. The confidence level is a percentage representation of the *p* value. Using the JMP software, the optimal value of the biocellulose conc. yield was calculated. A three-dimensional graph was created using STATISTICA 7.0 software^70,71^, in order to display the simultaneous impact of the three most significant independent factors on each response.

### Instrumental characterization of bacterial cellulose

FTIR: The chemical structures of BC membranes were analyzed by FTIR (IR, 8400 s Shimadzu, Japan), with the IR fingerprints recorded between 4000 and 400 cm^−1^ using transmittance modes. XRD: The overall crystalline phases of BC membranes were determined by XRD measurement on an (X Ray Diffractometer, Malvern Panalytical Empyrean, France). Radial scans of intensity were recorded at ambient conditions over scattering 2 angles from 5° to 80° with a step increment of 0.02°/s. SEM: The surface structure of BC membranes was investigated by scanning electron microscopy (SEM, Joel GSM-6610LV, Japan). The average diameter of bacterial cellulose fibers was measured by software *Image-J*.

### Instrumental characterization of SEDCFs

FTIR: The chemical structure of degraded CFs waste (SEDCFs) was analyzed by FTIR (IR, 8400 s Shimadzu, Japan) with the IR fingerprints recorded between 4000–400 cm^−1^ using transmittance modes. XRD: The overall crystalline phases of SEDCFs were determined by XRD measurement on a (X Ray Diffractometer, Malvern Panalytical Empyrean, France). Radial scans of intensity were recorded at ambient conditions over scattering two angles from 5° to 80° with a step increment of 0.02°/s. SEM: The surface structure of the purified SEDCFs was investigated by scanning electron microscopy (SEM, Joel GSM-6610LV, Japan).

### Preparation of HSP

Sugarcane bagasse was treated by two chemical treatments for pulping to remove the lignin content and then bleaching to obtain the pure cellulose which was used for HSP making according to the S.C.A standard, using a sheet former (S.C.A model-AB Lorentzen and Wettre). The HSP was prepared as reported by Atykyan et al.^56^ with minor modifications. Briefly, about 1.8 g of bleached sugarcane bagasse was homogenized with 5–7 L of tap water. After a homogenizing step, the suspension is separated through a screen. A sheet of 214 cm^2^ surface area and 165 mm in diameter is formed in the appliance, and then it is pressed for 4 min using a hydraulic press. The wet sheet is then collected on blotting paper, protected between two sheets. Drying of the prepared sheets is finished by using a rotary drum dryer for 2 h at 105 °C.

### Coating of HSP

The coating of HSP was carried out according to Vaithanomsat et al.^75^ with little modifications. SEDCFs were used as coating agent at different concentrations (1, 2 and 3% with respect to SEDCFs). Each concentration was prepared by homogenising it through ultra-sonication (750 wt, 20 KH, pulse 45, Amp 1) for 15 min to obtain a homogenous solution. About 20 ml of this solution was poured into the container (25X 20 cm) and the HSP was immersed in it for about 7 s, and then it was pressed between two sheets and oven dried at 45 °C until constant weight.

### Mechanical properties

Tensile strength testing is achieved utilizing a universal testing machine (LR10K; Lloyd Instruments, Fareham, UK) with a 100-N load cell at a constant crosshead speed of 2.5 cm/min in line with TAPPI (T494-06) standard method was used to determine the mechanical properties of coated HSP with different concentrations of SEDCFs. The gauge length is set at 10 cm and strips of 15 mm in width and 15 cm in length are used for the analysis. Each HSP thickness was determined by an electronic digital micrometer before the examination. Each test was performed by using 3 specimens, and the average of the results was recorded.

### Physical properties

#### *WVP (g.m*^*−1*^*.KPa*^*−1*^*.hr*^*−1*^*)*

The WVP of blank and coated HSP was evaluated according to the standard method ASTM E96-E80 with minor modifications. About 1.5 cm of blank and coated HSP with different concentrations of SEDCFs was sealed on the falcon tube (15 ml) containing 1 g of anhydrous calcium chloride. Plastic adhesive film was used to maintain the sample with the wide rim of the falcon tube to avoid air penetration. They were weighed and then placed in desiccators containing saturated potassium sulphate solution to get 95% relative humidity (RH) at 25 °C throughout the experiment. The weight of the falcon tube covered with film was monitored every day for a period of 10 days. The WVP of paper samples was calculated using the following Eq. (1):

1 $${\text{WVP}}\, = \,{\text{W}}\,{\text{x}}/{\text{t}}\,{\text{A}}\,\Delta {\text{P}}.$$

(W/t) = the slope of the plot between weight gain and time, x = the average thickness of the coated HSP, A = the permeation area, and ΔP = the partial water vapor pressure difference of the atmosphere in the cup and saturated sodium chloride solution corresponding to 0–95% RH.

#### Oil resistance barrier (ORB)

The oil resistance was measured according to the TAPPI (T-454 om-06) method. In this study, olive oil was used. the tested areas of the uncoated and coated HSP samples were placed under defined conditions of 25 °C and 50% RH; then, samples were placed in contact with a piece of white blank paper and 5 g of sand with a specified particle size (Sieve No. 30) on top of coated paper sheets; about (1.1 ml) of oil with soluble red dye saturated the sand sample. The time required for the oil to penetrate the samples was recorded to the nearest 10 s. The test of oil penetration was calculated as the average value of three measurements, where the resistance % was calculated as (the time required for the oil to penetrate the SEDCFs -coated HSP/the time required for the oil to penetrate the uncoated HSP) *100.

#### The efficacy of SEDCFs in heavy metals removal from aqueous solutions

The efficacy of SEDCFs sludge on different heavy metals removal was evaluated in batch conditions against four metal ions including FeSO_4_.7H_2_O, CrCl_3_.6H_2_O, CuCl_2_.2H_2_O and CoCl_2_.6H_2_O. The metal removal experiment was conducted in a 250 ml Erlenmeyer flask containing 100 mL of metal solution cocktail (0.1 g/L final concentration of each metal) and 1 g of the SEDCFs sludge. The flasks were incubated under shaking (200 rpm) at 30 °C, for 48 h., then the SEDCFs residue was separated by centrifugation and the residual metals concentrations were determined in the supernatant using atomic absorption spectrometry (Perkin Elmer A Annalist 100 spectrophotometer equipped with an air-acetylene burner). All the experiments were conducted in triplicate, and the average results are reported and compared to positive controls containing 0.1 g/L final concentration of each metal. The concentrations of the four metals were also evaluated in the SEDCFs fraction suspended in 100 ml of water and cultivated at the same experiment conditions (negative control). The sorption capacity q (mg/g) of the CFs sludge was calculated using the following equation: $\mathbf{q}=\frac{\mathrm{C}0-\mathrm{Ct}}{\mathrm{W}}\mathrm{V}$ where C0 is the initial metal concentration (mg/L) and Ct is the remaining metal concentration (mg/L) at the time t (h), V is the volume of solution (L), and W is the sorbent amount (g).

## Data Availability

All data produced during this study are included in this published article.
