# Author Correction: Fabrication of biodegradable chicken feathers into ecofriendly-functionalized biomaterials: characterization and bio-assessment study

**DOI:** 10.1038/s41598-022-26507-1

**Published:** 2022-12-21

**Authors:** Doaa A. Goda, Mohamed A. Diab, Hamada El‑Gendi, Elbadawy A. Kamoun, Nadia A. Soliman, Ahmed K. Saleh

**Affiliations:** 1 Bioprocess Development Department, Genetic Engineering and Biotechnology Research Institute (GEBRI), City of Scientific Research and Technological Applications (SRTA-City), New Borg El-Arab City, Universities and Research Institutes Zone, P.O. 21934, Alexandria, Egypt; 2 Cellulose and Paper Department, National Research Centre, 33 El‑Bohouth St., Dokki, P.O. 12622, Giza, Egypt; 3 Nanotechnology Research Center (NTRC), The British University in Egypt (BUE), P.O. 11837, El-Sherouk City, Cairo Egypt; 4 Polymeric Materials Research Dep. Advanced Technology and New Materials Research Institute (ATNMRI), City of Scientific Research and Technological Applications (SRTA-City), New Borg Al-Arab City, Alexandria, 21934 Egypt

Correction to: *Scientific Reports* https://doi.org/10.1038/s41598-022-23057-4, published online 31 October 2022

The original version of this Article omitted an affiliation for Elbadawy A. Kamoun. The correct affiliations are listed below.

Nanotechnology Research Center (NTRC), The British University in Egypt (BUE), El-Sherouk City, P.O. 11837, Cairo, Egypt

Polymeric Materials Research Dep. Advanced Technology and New Materials Research Institute (ATNMRI), City of Scientific Research and Technological Applications (SRTA-City), New Borg Al-Arab City 21934, Alexandria, Egypt.

The original Article has been corrected.

